# Genome Sequence of Bacillus megaterium O1, a Saponin-Degrading Bacterium

**DOI:** 10.1128/MRA.00524-20

**Published:** 2020-10-01

**Authors:** Cassandra E. Overney, Jean J. Huang

**Affiliations:** a Franklin W. Olin College of Engineering, Needham, Massachusetts, USA; University of Maryland School of Medicine

## Abstract

Bacillus megaterium strain O1 was isolated from a soapnut (Sapindus saponaria) surface and degrades *Quillaja* saponin as a sole carbon source. We report the draft genome sequence of B. megaterium O1, which has an estimated size of 5.1 Mb. Study of this isolate will provide insight into mechanisms of saponin degradation.

## ANNOUNCEMENT

Though saponin-degrading enzymes have been identified in fungi and plants (1), few enzymes have been identified from bacteria. Within the human gut microbiota, saponin-degrading microbes enable host access to the anti-inflammatory, antiallergy, and antitumor effects of various traditional Chinese medicinal herbs (2, 3). Saponin degradation is important for livestock animal feed, such as tea seed meal, which can be less toxic if saponin is removed (4). We isolated Bacillus megaterium O1 from a soapnut (Sapindus saponaria) surface through enrichment with 1% *Quillaja* saponin (Catalog of Chemical Suppliers [CAS] number 74499233) as the sole carbon source on a plate containing freshwater mineral salts agar (5). A colony of the isolate was purified by successive streak plating onto fresh agar medium. B. megaterium O1 grew in liquid culture with 1% saponin with a doubling time of 1.5 ± 0.02 h. Due to the potential application of Bacillus megaterium as a probiotic (6, 7), the study of this strain can provide insight into how saponin degradation occurs or may be applied.

Genomic DNA was extracted from pelleted cells from liquid culture using the PowerLyzer PowerSoil DNA isolation kit (Mo Bio, Germantown, MD) and quantified using the Qubit double-stranded DNA (dsDNA) high-sensitivity (HS) assay kit (Life Technologies). The library was prepared using the Nextera DNA Flex library preparation kit (Illumina). Genome sequencing was carried out using an Illumina NovaSeq instrument at Molecular Research DNA (Shallowater, TX) with 2 × 250-nucleotide paired-end reads. Genome assembly of 5 million paired reads was performed using NGEN V15 (DNAStar). Default parameters were used unless otherwise noted. The genome sequence of B. megaterium O1 has 62 contigs and consists of 5,102,251 bp with an average GC content of 37.99% and an *N*_50_ contig size of 4,132,152 bp with 100× coverage. Genome annotation was carried out using the NCBI Prokaryotic Genome Annotation Pipeline (GeneMarkS-2 v. 4.11) (8). Of the 5,582 predicted genes in the genome, 95.93% encode proteins, 72.23% of which have a predicted function.

The O1 genome was searched for homologs to known genes for saponin-degrading enzymes. From a blastp search (9, 10) of all 91 saponin hydrolase protein sequences in the NCBI database to the O1 genome, genes for two known saponin-degrading enzymes both pointed to homology with the O1 gene 6-phospho-β-glucosidase (GenBank accession number NLR44478.1), which was 33% similar to a β-glucosidase of Costus speciosus (11) (Q42707.1) with an E value of 3.00E-69 and 27% similar to a β-glucosidase of Trichoderma viride (1) (QBZ28529.1) with an E value of 1.00E-33.

Within the O1 genome, the potential saponin-degrading gene 6-phospho-β-glucosidase (GenBank accession number NLR44478.1) is located in a cluster of genes related to carbohydrate metabolism and amino acid transport, including a PTS system β-glucoside-specific IIC component (NLR44477.1) and a GntR family transcriptional regulator of *bglA* (NLR44476.1) that could be involved in 6-phospho-β-glucosidase gene regulation. Further study of strain O1 will provide insights into the capabilities of the bacterium and its enzymes involved in saponin degradation and their regulation.

### Data availability.

The GenBank accession number for this genome is JABAKC000000000 (BioProject number PRJNA622595). The SRA accession number is SRR11514001. The JGI genome number is 2830408603.
